# Toward visualized pathophenotyping: a multimodal imaging-guided conceptual framework for chronic low back pain

**DOI:** 10.3389/fmed.2026.1932798

**Published:** 2026-09-11

**Authors:** Peijie You, Ming Fang, Zheng Yan, Suliang Xu, Jiale Zhang, Guanyi Gong

**Affiliations:** 1 Suzhou TCM Hospital Affiliated to Nanjing University of Chinese Medicine, Suzhou, China; 2 Suzhou Guangji Hospital, The Affiliated Guangji Hospital of Soochow University, Suzhou, China; 3 China Science and Technology Development Center for Chinese Medicine, Beijing, China

**Keywords:** acupotomy, chronic low back pain, infrared thermography, shear wave elastography, ultrasound-guided needle-knife therapy, visualized pathophenotyping

## Abstract

Chronic low back pain (CLBP) remains a leading cause of disability, yet many current interventions are limited by operator dependence and an inability to target deep pathophysiological drivers. This Perspective proposes Visualized Pathophenotyping, a conceptual precision paradigm that integrates multimodal imaging to characterize patient-specific deep structural and perfusion abnormalities and guide targeted interventions. The framework comprises three pillars: (1) precise assessment using shear wave elastography and infrared thermography or laser Doppler flowmetry to define mechanical and ischemic phenotypes; (2) ultrasound-guided deep mechanical stimulation for accurate targeting of fascial adhesions and myofascial trigger points; and (3) phenotype-specific intelligent monitoring with virtual/augmented reality and wearable AI to sustain tissue remodeling and prevent recurrence. Emerging evidence from randomized trials and meta-analyses suggests that visualization-guided approaches yield superior short-term pain relief and functional improvement compared with blind techniques. Digital therapeutics may enhance adherence and some functional outcomes, although broader biopsychosocial effects remain to be confirmed. By shifting CLBP management from operator-dependent procedures toward objective, data-driven care, this hypothesis-generating framework may reduce recurrence, minimize opioid reliance, and improve scalability, pending validation through prospective multicenter trials of diagnostic accuracy, clinical utility, and cost-effectiveness.

## Introduction

Chronic low back pain (CLBP) is a leading cause of disability, affecting approximately 619 million people in 2020 and projected to reach 843 million by 2050 due to population growth and ageing (1). This rising burden positions CLBP as a major public health challenge, contributing substantially to years lived with disability and escalating healthcare costs (2). Despite a wide array of available treatments, conventional approaches, such as pharmacotherapy, superficial physical modalities, and blind dry needling, continue to yield high recurrence rates, often exceeding 50% within one year (3, 4). This high recurrence largely reflects a failure to target the deep pathophysiological drivers of CLBP. Persistent pain frequently originates from “invisible” deep structures: periosteal adhesions at fascial attachments, deep myofascial trigger points in muscles such as the multifidus and quadratus lumborum, and localized perfusion deficits causing ischemic hypersensitivity (5–7). These lesions can perpetuate a cycle of chronic inflammation and mechanical stress that may secondarily contribute to central sensitization (8). Conventional therapies that primarily address superficial symptoms therefore leave these deeper pathological substrates incompletely addressed. The proposed model considers CLBP a multifactorial condition in which peripheral nociceptive sources (periosteal adhesions, deep myofascial trigger points, and perfusion deficits) interact with central nervous system alterations. Visualization-guided interventions primarily target the peripheral substrates, with the expectation that reducing peripheral drive may secondarily modulate central sensitization. This view does not exclude primary central mechanisms but prioritizes identifiable peripheral pathology as an actionable entry point for precision care (9, 10). For instance, a recent double-blind randomized controlled trial that applied dry needling to the lumbar multifidus and erector spinae at the most painful side and spinal level found that, one week after treatment, resting erector spinae stiffness measured by shear wave elastography was significantly lower in the dry needling group than in the sham group (mean change −13.5% vs. +6.0%; adjusted between-group difference −1.3 kPa, *p* = 0.019). Pain (Numeric Pain Rating Scale) and Oswestry Disability Index (ODI) improved within both groups, but only the global rating of change and lumbar flexion range of motion significantly favored dry needling over sham (11). Further supporting this, a 2022 prospective randomized trial (12) evaluating a dry needling program based on the Five Regulatory Systems concept (a traditional Chinese medicine framework that coordinates regulation of the nervous, circulatory, endocrine, immune, and musculoskeletal systems to restore systemic balance) in patients with CLBP due to L5-S1 discopathy found significant reductions in pain intensity, as measured by the visual analogue scale (VAS; decrease of up to 6.45 points), and improvements in functional status, as measured by the ODI (decrease of up to 18.9 points), compared with sham therapy, with effects sustained at 3-month follow-up. Both changes exceeded commonly cited minimal clinically important differences of approximately 2 points for VAS and 10 points for ODI (13). Meanwhile, ultrasound-guided needle-knife therapy targeting deep ligamentum flavum or bony enthesopathies has achieved rapid pain relief and functional restoration in refractory cases, outperforming blind techniques (14, 15).

Despite these benefits, clinical translation remains constrained by heavy reliance on operator-dependent palpation. Blind procedures frequently fail to reach deep targets, with needles remaining in superficial fascia rather than periosteal adhesions or deep hypertonic zones. This results in inconsistent therapeutic depth, variable local twitch responses, incomplete adhesion release, and high outcome heterogeneity (16). Meta-analyses confirm that although deep stimulation is effective, outcomes vary widely due to technique variability and lack of standardization, limiting scalability and reproducibility (16–18).

The pursuit of precision in pain management has recently reached the central nervous system: a 2026 Nature study (19) demonstrated that targeted chemogenetic modulation of cortical pain circuits can replicate opioid analgesia without addiction. Yet this central advance underscores a parallel peripheral gap: the deep pathological substrates of CLBP—periosteal adhesions, myofascial trigger points, and perfusion deficits—remain invisible to conventional palpation.

We therefore introduce the concept of Visualized Pathophenotyping, defined as the use of multimodal imaging to render deep structural and perfusion abnormalities into quantifiable, patient-specific digital maps that can serve as the anatomical and physiological basis for subsequent intervention. This concept reframes CLBP not as a uniform syndrome but as a spectrum of potentially identifiable pathophysiological subtypes (mechanical, ischemic, or mixed), each of which may require a distinct therapeutic strategy. The framework is presented as a conceptual model intended to guide future research rather than as an established evidence-based clinical pathway.

## Visualization-enabled precise assessment: revealing and quantifying deep pathological roots

Traditional palpation and clinical examination in CLBP are inherently subjective, often failing to delineate deep pathological structures such as bony attachment adhesions (e.g., enthesopathies at the iliac crest or spinous processes), deep myofascial trigger points, and microvascular perfusion deficits that perpetuate pain cycles (20). These “invisible” roots contribute to neuromuscular imbalances and chronic sensitization, yet conventional diagnostics like radiographs or standard MRI lack the resolution or real-time capability to quantify them dynamically. Digital imaging modalities, as foundational elements of this visualization-centric framework, address this gap by providing objective, quantifiable visualization, converting qualitative assessments into data-driven “pathology maps” that guide subsequent interventions. We propose that CLBP is better understood as a spectrum of distinct deep pathophysiological phenotypes, each requiring a specific therapeutic logic. Shear wave elastography (SWE) and infrared thermography (IRT), used in combination, provide the objective basis for this reclassification.

SWE exemplifies this by measuring tissue stiffness in real time (21). However, SWE quantifies tissue stiffness—a biomechanical property—not tissue architecture. Elevated shear modulus may reflect fascial contracture, but it can equally reflect muscle hypertonicity, inflammatory edema, or transient postural effects (22). Therefore, in the context of CLBP phenotyping, SWE-derived stiffness is best regarded as an indirect, nonspecific biomarker of mechanical pathology that requires correlation with clinical findings and, ideally, real-time ultrasound visualization to infer the presence of fascial adhesion or contracture.

Within this interpretive framework, emerging evidence supports the clinical utility of SWE in CLBP. A 2024 investigation (23) using SWE revealed significantly elevated stiffness in the thoracolumbar fascia and multifidus muscle in patients with chronic nonspecific low back pain, with shear modulus values higher than controls at the L4–5 level. Stiffness values showed moderate correlations with pain intensity (*r* = 0.57–0.65). A separate reliability study (24) confirmed SWE's excellent intra-rater reproducibility (ICC ≥0.90 for stiffness and ≥0.80 for thickness) for assessing lumbar fascia properties in both healthy and CLBP cohorts. These findings establish SWE as a feasible and reproducible tool for quantifying mechanical pathology in CLBP, supporting its role in defining the hyperrigid phenotype when interpreted alongside clinical indicators of mechanical restriction.

Complementing structural mapping, infrared thermography (IRT) and laser Doppler flowmetry (LDF) visualize deep physiological status, specifically microvascular perfusion changes critical to ischemic-driven CLBP (25). A 2022 systematic review (26) highlighted IRT as a valuable non-invasive supplementary tool in musculoskeletal disorders and rehabilitation, particularly for evaluating therapeutic effects of physical interventions on inflammation and tissue recovery. IRT detects asymmetric thermal patterns indicative of inflammation or hypoperfusion, with research demonstrating diagnostic accuracy of 80% for lumbosacral radicular pain using temperature differentials (optimal *Δ*T range: 0.8°C–2.2°C), which increases to 84% when combined with clinical assessment (27). A 2025 study (28) on digital infrared thermography in degenerative spinal disorders demonstrated that hypothermic patterns in compressed nerve root regions (e.g., due to disc herniation or spinal stenosis) indicate reduced blood flow and vascular insufficiency, highlighting IRT's role in identifying deep ischemic “hotspots” in CLBP mechanisms. LDF, as applied in a randomized, placebo-controlled trial, demonstrated that myofascial release techniques significantly improved lumbar myofascial blood flow as measured by laser Doppler flowmetry, with increases of 31.6% immediately post-treatment and 48.7% at follow-up compared to placebo, supporting the role of targeted interventions in alleviating hypoxia-induced inflammation (29). Together, these modalities define the “hypoperfusion phenotype” characterized by ischemic inflammation, metabolic irritation, and chemically driven sensitization. For these patients, the therapeutic priority shifts from mechanical disruption to perfusion restoration and metabolic modulation.

**Figure 1:**
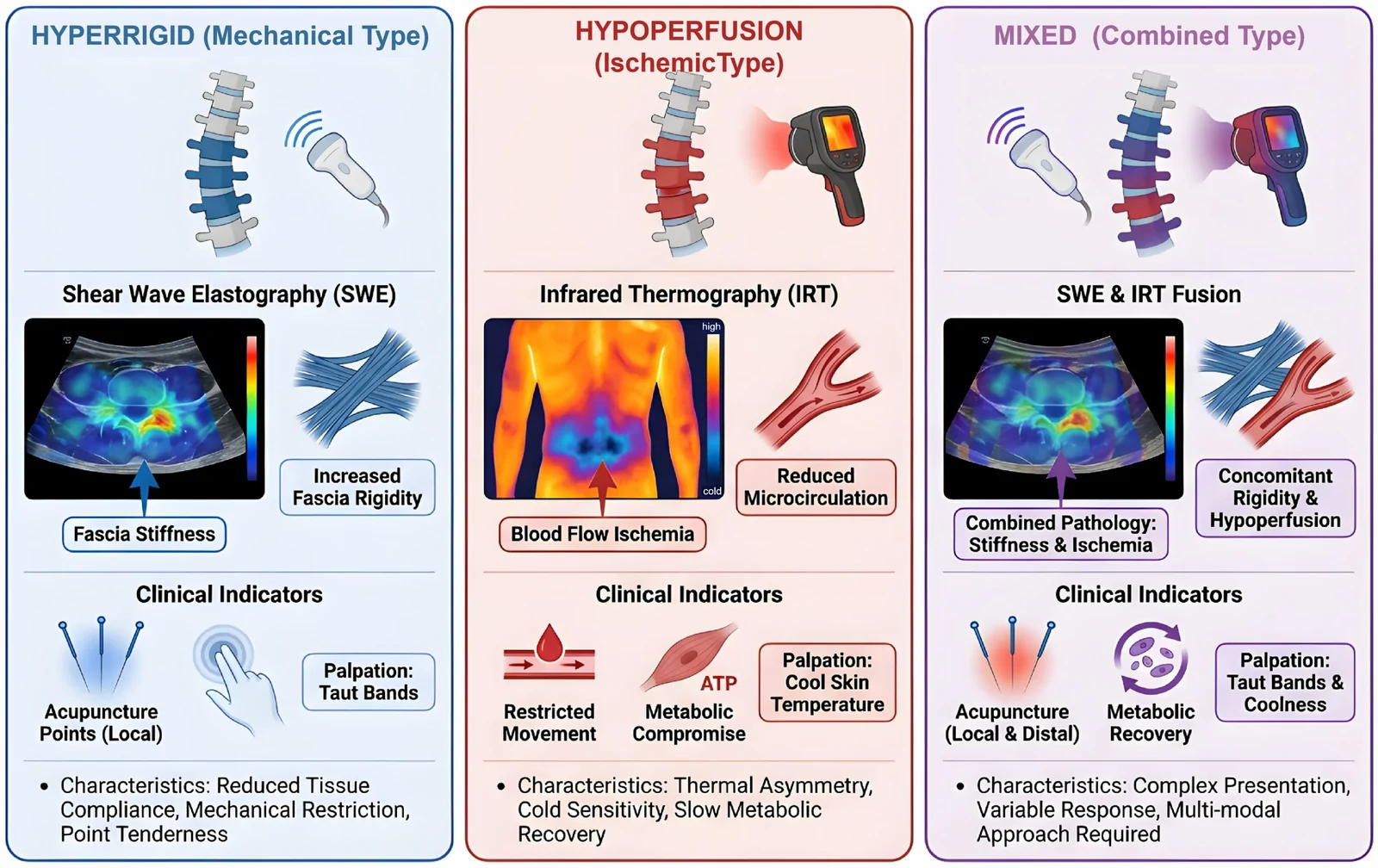
Visualized pathophenotyping spectrum in chronic low back pain. Multimodal imaging (SWE and IRT) enables objective classification of CLBP into three pathophysiological phenotypes: hyperrigid (mechanical, characterized by increased tissue stiffness on SWE, interpreted as an indirect marker of fascial or myofascial pathology), hypoperfusion (ischemic, inflammation-driven, characterized by reduced microcirculation), and mixed (combined mechanical and ischemic components). Each phenotype is associated with distinct clinical indicators and therapeutic priorities: mechanical release for hyperrigid, perfusion restoration for hypoperfusion, and combined approach for mixed presentations. The bottom panel illustrates how phenotype-guided assessment converges toward precision intervention, shifting CLBP management from empirical, one-size-fits-all approaches to mechanism-guided, patient-specific care.

We propose three preliminary conceptual phenotypes based on multimodal imaging. The hyperrigid phenotype is defined by elevated SWE stiffness values in the thoracolumbar fascia or multifidus that exceed reported normative ranges at the vertebral levels examined, for example L4-5, together with clinical indicators of mechanical restriction. The hypoperfusion phenotype is defined by asymmetric hypothermic patterns on infrared thermography or reduced microvascular flow on laser Doppler flowmetry, consistent with ischemic or inflammatory changes. The mixed phenotype is defined by concurrent elevation of both stiffness and hypoperfusion markers. These categories are intended as a preliminary conceptual framework requiring prospective validation of diagnostic thresholds, phenotypic stability, and relationship to central sensitization; mixed presentations are acknowledged but currently incompletely characterized. This phenotyping framework shifts the fundamental question of CLBP management from “which needle technique works best?” to “which phenotype does this patient have, and what intervention matches it?” This distinction lies at the heart of precision musculoskeletal medicine. Figure 1 provides a visual summary of this three-phenotype classification—hyperrigid, hypoperfusion, and mixed—integrating imaging findings, clinical indicators, and corresponding therapeutic logic. The spectrum framework illustrates how SWE-defined mechanical pathology and IRT-defined ischemic pathology can be objectively distinguished, and where they overlap in mixed presentations. Table 1 highlights selected studies demonstrating these distinct pathophysiological profiles in low back pain cohorts.

**Table 1:** Key randomized trials demonstrating deep pathophysiological changes with targeted interventions in low back pain.

Intervention/Measure	Key Physiological/Clinical Outcome	Change/Effect (Details)	Time Point/Comparison	Significance/Notes	Reference
Dry Needling (using SWE)	Resting erector spinae muscle stiffness	Reduction in DN group vs. increase in sham (∼ −13.5% vs. +6%)	1-week post-treatment	Significant reduction; supports deep muscle normalization	Koppenhaver et al. (11)
Myofascial Release (lumbar)	Lumbar microcirculation (blood flow)	+31.6% immediately; +48.7% at follow-up	Post-treatment & follow-up vs. placebo	*p* < 0.0001; links to hypoxia-inflammation relief	Brandl et al. (29)
US-Guided Needle-Knife (refractory NSLBP)	NRS/ODI/JOA scores; Multifidus muscle thickness	Significant improvements in pain/function; multifidus thickness ↑	2–12 weeks; vs. usual care	Safe, shorter treatment; deep structural recovery	Li et al. (15)
Needle-Knife (lumbar disc herniation)	VAS, JOA, ODI	MD −1.44 (VAS), +2.93–3.73 (JOA), −4.93 (ODI)	Overall vs. traditional	Superior efficacy; supports periosteal/deep release	Zhang et al. (17)

SWE, shear wave elastography; NSLBP, nonspecific low back pain; DN, dry needling; NRS, numeric rating scale; JOA, Japanese Orthopaedic Association score. These trials highlight objective deep changes (stiffness, perfusion, muscle morphology) that are measurable with visualization/targeted approaches and are consistent with, though not pathognomonic for, specific deep tissue pathology.

## Visualization-guided interventions: ensuring direct access to deep pain points

Real-time ultrasound imaging transforms the pathology map from a diagnostic reference into an intraoperative navigation system, ensuring that mechanical stimuli reach the precise anatomical locations identified during assessment, rather than relying on probabilistic blind placement. This shift—from “searching” to “navigating”—is the operational core of precision delivery in CLBP management.

**Figure 2:**
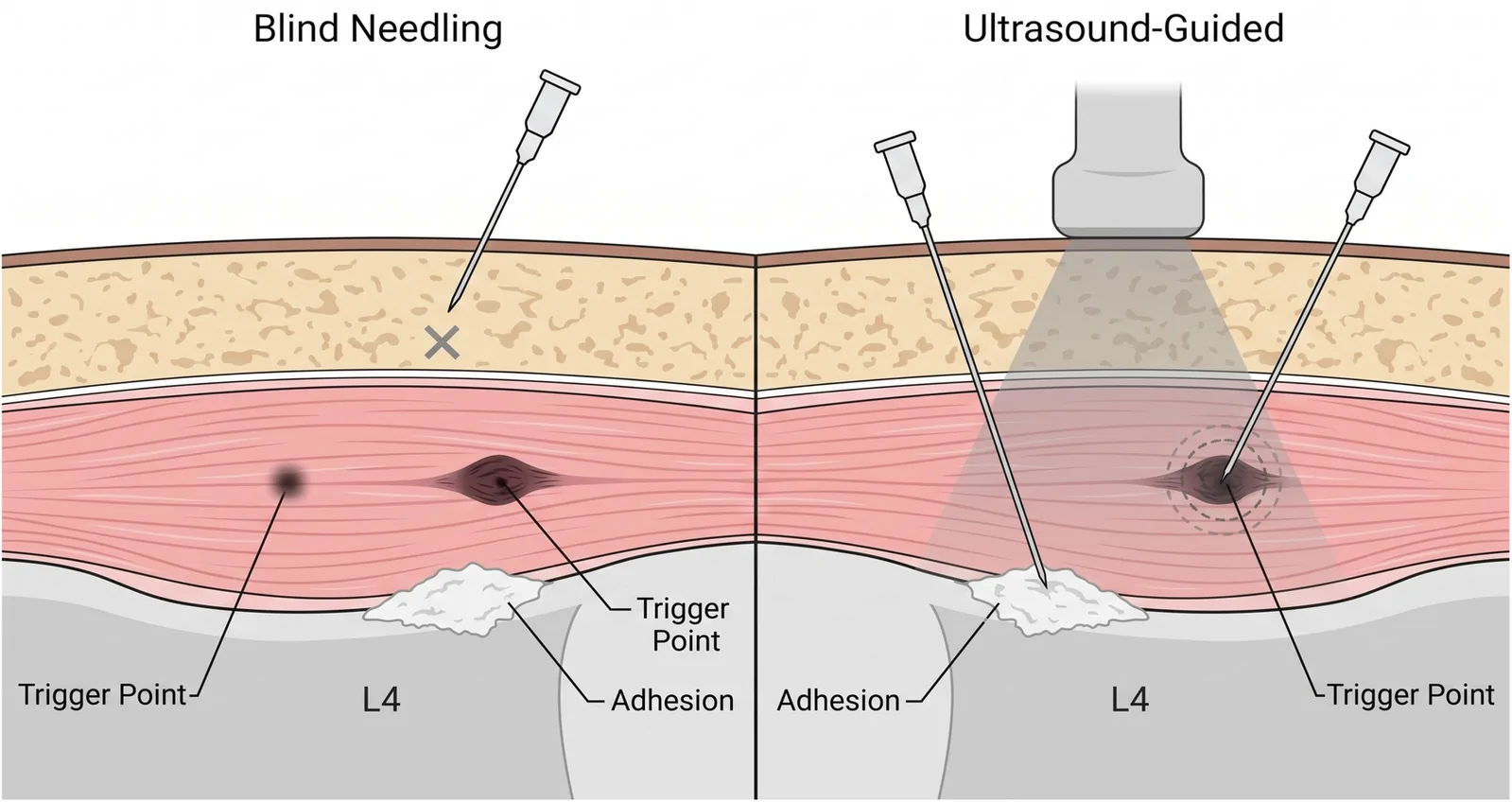
Blind vs. ultrasound-guided needling for deep pathological targets. Sagittal split-view comparison of the lumbar region. Left: blind needling terminates in subcutaneous fat, missing the periosteal adhesion and myofascial trigger point. Right: ultrasound guidance enables precise needle placement, with one tip releasing the adhesion and another eliciting a local twitch response (concentric ripples) within the trigger point. The gray wedge indicates the ultrasound scanning plane.

Ultrasound-guided needle-knife therapy (US-NKT) and dynamic dry needling exemplify this precision, with high-resolution probes visualizing needle trajectories in real time, enabling clinicians to target periosteal hyperplasias, deep trigger points in the quadratus lumborum, or fibrotic bands within the multifidus—structures that are readily visualized on SWE as discrete hyperrigid zones of elevated stiffness but are entirely occult to palpation (30). A 2025 systematic review and meta-analysis (14) of US-NKT for spinal pain disorders reported superior short-term pain relief (SMD ranging from −1.11 at 1 week to −1.74 at 1 month) and functional improvement (strongest SMD −0.92 at 1 week, significant across time points) compared to conventional NKT or controls, with adverse event rates in the US-NKT group at 4.6%. A 2026 prospective randomized trial (31) directly compared ultrasound-guided deep vs. superficial dry needling in patients with myofascial pain syndrome of the upper trapezius. Under real-time ultrasound guidance, the deep dry needling group received needle insertion directly into the myofascial trigger points, whereas the superficial group received needling only at the subcutaneous deep fascia. At one-week follow-up the deep group demonstrated significantly greater improvements in pain (NPRS), disability (Neck Bournemouth Questionnaire, NBQ; Neck Disability Index, NDI), pressure pain threshold, and cervical range of motion. Although this study supports the importance of depth and precision in myofascial needling, evidence derived from upper-trapezius myofascial pain syndrome cannot be directly extrapolated to lumbar musculature in CLBP; direct validation in lumbar-specific cohorts is needed. Figure 2 contrasts blind needling with ultrasound-guided intervention, demonstrating how real-time visualization ensures precise needle placement at deep periosteal adhesions and myofascial trigger points, thereby overcoming the limitations of operator-dependent techniques.

The evidence consistently indicates that visualization guidance enhances both the accuracy and the consistency of deep mechanical interventions. By confirming needle position relative to the target before, during, and after delivery, ultrasound reduces the variability inherent in palpation-dependent placement and ensures that the therapeutic stimulus reaches the structure identified on pre-procedural imaging. Table 2 summarizes key pooled effect sizes from high-quality evidence supporting this precision paradigm. In summary, visualization guidance does not replace clinical judgment; it informs and verifies it. It converts an experience-dependent procedure prone to incomplete release and off-target placement into a target-confirmed intervention with enhanced precision, improved safety, and reproducible outcomes across operators.

**Table 2:** Meta-analyses of visualization-guided and digital interventions for chronic low back pain or related spinal disorders.

Intervention	Key Outcomes	Effect Size (95% CI)	Time Point/Population	Heterogeneity (I^2^/Certainty)	Main Findings & Implications for Deep Targeting	Reference
Ultrasound-Guided Needle-Knife Therapy (US-NKT)	Pain Reduction	SMD −1.11 (−1.42 to −0.79)	1 week	73%	Superior short-term; favorable safety (AE 4.6%)	Kim et al. (14)
	Pain Reduction	SMD −1.74 (−2.50 to −0.98)	1 month	95%	Strongest effect; vs. conventional NKT/controls	Kim et al. (14)
	Physical Function Improvement	SMD −0.92 (−1.42 to −0.42)	1 week (strongest)	71%	Significant across time points	Kim et al. (14)
Dry Needling (combined/other therapies)	Pain Intensity	SMD −0.42 (−0.79 to −0.05)	Post-intervention	–	Effective for myofascial CLBP pain relief	Lara-Palomo et al. (18)
	Pain Intensity	SMD −0.99 (−1.61 to −0.37)	Short-term follow-up	–	Recommended for short-term; no disability benefit	Lara-Palomo et al. (18)
Virtual Reality (VR)	Pain (VAS)	MD −1.69 (−2.47 to −0.88)	Overall	Moderate-high	Significant reduction; immersive/high-frequency better	Huang & Chen (33)
	Disability (ODI)	MD −5.92 (−7.97 to −3.86)	Overall	–	Improved function & kinesiophobia	Huang & Chen (33)
Mobile Apps + Rehabilitation	Pain Intensity	MD −0.58 (−1.00 to −0.17)	Long-term (mixed LBP)	Moderate certainty	Slight long-term benefit vs. rehab alone	Ferrero et al. (34)

SMD, standardized mean difference (negative favors intervention); MD, mean difference; AE, adverse events. Heterogeneity high in some US-NKT outcomes; sustained benefits beyond 3 months inconclusive for US-NKT. Data support visualization-guided deep interventions for superior short-term precision in CLBP.

## Phenotype-specific post-intervention maintenance: achieving durable remodeling and recurrence prevention

Because direct evidence matching specific maintenance interventions to imaging-defined CLBP phenotypes is currently lacking, the following strategies should be regarded as hypothesis-based rather than established treatment protocols. Durable CLBP resolution requires more than successful initial intervention—it demands that the underlying pathophysiological substrate be systematically remodeled to prevent re-emergence of the treated lesion. This principle follows directly from the phenotyping framework: a hyperrigid patient whose fascial compliance has been restored remains susceptible to re-contracture if mechanical loading patterns are not retrained; a hypoperfusion patient whose microcirculation has improved remains vulnerable to ischemic recurrence if cardiovascular conditioning is neglected. We therefore argue that post-intervention maintenance should be prescribed not as a generic rehabilitation protocol, but as a phenotype-specific strategy derived from the same SWE/IRT profile that guided the initial intervention.

For patients with the hyperrigid phenotype, the primary maintenance objective is sustained tissue compliance and neuromuscular control. Dynamic stretching—targeting the thoracolumbar fascia and multifidus—combined with proprioceptive training (core stabilization, segmental motor control) addresses the mechanical memory that predisposes to re-contracture. Virtual reality (VR) and augmented reality (AR) are particularly suited to this phenotype: VR immerses patients in simulated environments to practice controlled spinal movements with real-time postural feedback (32), while AR overlays movement patterns onto the patient's physical environment, guiding home-based stretching with precision. A 2025 meta-analysis (33) demonstrated significant functional improvements with VR in CLBP (ODI MD −5.92) compared to standard exercises. Critically, for the hyperrigid phenotype, VR/AR functions as a mechanical retraining tool—not distraction or analgesia, but targeted motor relearning informed by the pre-treatment stiffness map.

For patients with the hypoperfusion phenotype, the maintenance logic diverges. Here, the therapeutic priority is sustained aerobic conditioning and microcirculatory promotion. Wearable devices—specifically those integrating heart rate, step count, and sleep quality monitoring—track cardiovascular engagement and detect sedentary patterns that predict ischemic relapse (34). AI algorithms analyze these data streams not to “close a loop” toward automated re-treatment, but to serve two clearly defined functions: (1) an early warning system that flags sustained reductions in physical activity or deteriorations in sleep-derived recovery metrics (35, 36), prompting timely clinical review; and (2) an adherence coach that delivers personalized motivational feedback and adjusts daily activity targets based on individual baseline capacity. For hypoperfusion patients, this wearable-AI ecosystem monitors the physiological substrate of recurrence, not as a proxy for imaging, but as a real-world indicator of microvascular health status between clinic visits.

## Challenges

Despite the promising potential of the Visualized Pathophenotyping framework, several challenges must be addressed to facilitate its clinical translation and widespread adoption. First, the integration of multimodal imaging (SWE, IRT, and LDF) requires standardized protocols for data acquisition, processing, and interpretation. Variability in equipment, operator technique, and environmental factors (e.g., room temperature for IRT) can affect reliability and comparability across settings (37, 38). While SWE and IRT have demonstrated good intra-rater reliability in recent studies, large-scale validation of combined modalities for phenotype classification remains limited. Second, equipment costs and accessibility pose significant barriers, particularly in resource-limited settings. High-resolution ultrasound systems and wearable AI platforms may not be readily available in primary care or community clinics, potentially exacerbating health disparities (39, 40). Third, operator training represents a critical bottleneck. Effective implementation demands proficiency in imaging interpretation, ultrasound-guided interventions, and phenotype-specific treatment planning—skills that extend beyond traditional palpation-based training (41). Fourth, data privacy, security, and ethical concerns surrounding continuous wearable monitoring and AI-driven decision support must be rigorously addressed to maintain patient trust (42).

Long-term evidence also remains insufficient. Although short-term benefits of visualization-guided interventions are well-documented, data on sustained recurrence prevention, cost-effectiveness, and impact on opioid reduction are limited. The stability of phenotypes over time and their interaction with psychosocial factors require further elucidation. Additionally, the definition and clinical significance of mixed phenotypes—in which mechanical and ischemic components co-exist—remain unclear, as no validated criteria currently exist to classify or quantify their relative contributions. These limitations underscore the need for prospective validation before clinical adoption.

## Future directions

Prospective multicenter trials are needed to test the diagnostic accuracy of the imaging-based phenotypes, the incremental value of phenotype-guided vs. standard care, and the durability of phenotype-specific maintenance strategies. To overcome these challenges and realize the full potential of this precision paradigm, several research priorities emerge. Large-scale, multicenter randomized controlled trials are essential to evaluate the clinical effectiveness, cost-effectiveness, and long-term outcomes of the complete visualization-centric precision framework compared with standard care. These trials should incorporate diverse populations to assess generalizability and include health economic analyses. Prospective longitudinal studies are needed to validate the stability, predictive value, and treatment responsiveness of SWE/IRT/LDF-defined phenotypes, including exploration of mixed phenotypes and their overlap with central sensitization mechanisms.

Technological advancement should focus on developing integrated, user-friendly platforms that combine multimodal imaging, real-time ultrasound navigation, and AI-powered wearable feedback into seamless clinical workflows. Research into low-cost, portable imaging solutions and standardized training curricula (e.g., simulation-based programs) will enhance accessibility. Additionally, implementation science studies should investigate optimal strategies for embedding this framework into primary care and multidisciplinary settings, addressing barriers related to workflow, reimbursement, and clinician acceptance.

Finally, advancing the biopsychosocial integration of the model—through hybrid interventions combining visualization-guided mechanical therapies with digital psychoeducation, biofeedback, or avatar-based approaches—could further enhance self-efficacy and long-term adherence. By prioritizing these directions through multidisciplinary collaboration among clinicians, engineers, data scientists, and policymakers, the Visualized Pathophenotyping framework can evolve from an innovative concept into a transformative standard of care for CLBP, ultimately reducing the global burden of this disabling condition.

## Data Availability

The original contributions presented in the study are included in the article/Supplementary Material, further inquiries can be directed to the corresponding author/s.
